# Deformation behavior of single-crystal magnesium during Nano-ECAP simulation

**DOI:** 10.1016/j.heliyon.2022.e11837

**Published:** 2022-11-23

**Authors:** Amro H. Altoyuri, Junaidi Syarif, Zainuddin Sajuri

**Affiliations:** a Department of Mechanical and Nuclear Engineering, University of Sharjah, United Arab Emirates; b Sustainable Energy & Power Systems Research Center, University of Sharjah, United Arab Emirates; c Department of Mechanical and Manufacturing Engineering, Faculty of Engineering and Built Environment, Universiti Kebangsaan Malaysia, 43600 Bangi, Selangor, Malaysia

**Keywords:** Magnesium, ECAP, Twinning, Molecular dynamics, Severe plastic deformation

## Abstract

Molecular dynamics was applied to simulate ECAP of single-crystal magnesium at room temperature. Four samples with different orientations were processed, and the grain structure, grain fragmentation, slip systems, strain, and twin formation were analyzed. The initial orientation played a substantial role in the strain and deformation experienced by the samples during both stages of deformation. Compressions initially occurred before extrusion, and simple shear occurred in the deformation zone during extrusion. The samples nucleated a $\left\{10\bar{1}2\right\}$ tension twin during compression, and the tension twin grew to immediately cover the entire sample, effectively changing the orientation of the sample. Additionally, stacking faults acted as a precursor for the $\left\{10\bar{1}2\right\}$ tension twin. The strain was strongly correlated with the shear factor, that is, a high shear factor resulted in low strain. Moreover, discrepancy occurred between theoretical and actual shear strain due to two factors. First, theoretical shear is considered to be simple shear occurring entirely in the deformation zone; it does not consider the shear strain due to the normal stress in the compression phase. Second, deformation is considered to be homogenous and isotropic, and it does not take into account the initial grain orientation and the anisotropic nature of magnesium.

## 1 Introduction

Magnesium is the lightest structural metal because of its low density. Thus, magnesium is widely adopted in aerospace, automobile, and manufacturing industries, especially when weight is a limiting factor [1]. Magnesium alloys exhibit high specific strength and good heat dissipation and damping characteristics [2]. They are extensively used in biomedical applications due to their biocompatibility, and they are considered a promising alternative to orthopedic implants because of their biodegradability [3].

Notably, magnesium suffers from high wear and corrosion rates due to its low hardness and highly reactive nature. However, recent research on alloy design and microstructural control processes has improved the mechanical properties, corrosion resistance, and biocompatibility of magnesium [2]. For instance, equal-channel angular pressing (ECAP) is a severe plastic deformation process that is applied to improve the mechanical properties of magnesium alloys by enhancing the tensile strength, grain refinement, and texture development of the microstructure for various applications [4, 5, 6].

The hexagonal close-packed (HCP) structure of magnesium deforms in an anisotropic manner, resulting in different deformation under tension when compared to compression [7]. The $\left(0001\right)\;<11\bar{2}0>$ basal slip is the main slip system for magnesium. However, satisfying the Von-Mises criterion is difficult because having only two independent slip systems results in the activation of non-basal slip systems and twins [8], thereby resulting in different deformation and twining under various loading conditions. For example, the $\{10\bar{1}2\}\;<10\bar{1}1>$ tension twin is predominantly present during c-axis tension, and the $\{10\bar{1}1\}\;<10\bar{1}2>$ compression twin is predominantly present during c-axis compression.

• Many studies have attempted to explain the mechanism of twin deformation under simple tension or compression. Twin deformation is commonly reported in magnesium, and twinning is widely explained by a series of slips and atomic shuffling [9, 10, 11, 12, 13]. However, the underlying mechanism of reshuffling remains unexplained in terms of kinetics and energetics. Alternatively, in a recent study, *Zhang et al.* proposed a different mechanism for the formation of the $\{10\bar{1}2\}\;<10\bar{1}1>$ tension twin, in which a series of stacking faults is used as a precursor to nucleate the twin [14].

Therefore, further research is required to elucidate the deformation and twinning behavior of magnesium during ECAP, which includes compression and shear deformation. In this study, molecular dynamics (MD) simulations were performed to elucidate how different initial orientations influence the deformation characteristics of single-crystal magnesium during one-pass ECAP at room temperature. The grain structure, grain fragmentation, dislocations, slip systems, strain, and twin formation during ECAP were analyzed and evaluated.

## 2 Methodology

**Figure 1:**
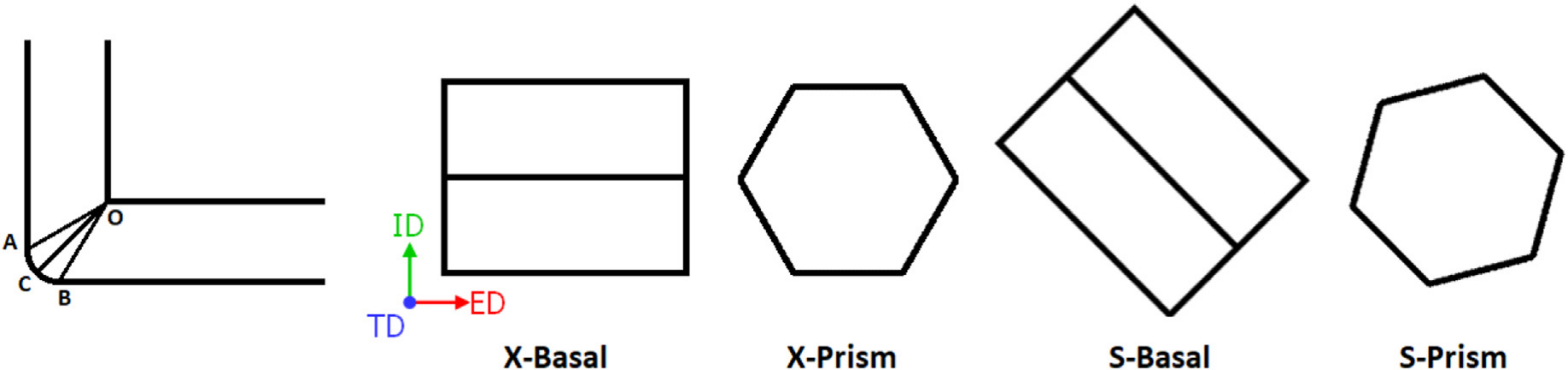
The geometry of the ECAP die and the crystal orientation of each sample with respect to the die.

The simulation was performed using the large-scale atomic/molecular massively parallel simulator (LAMMPS) [15], a molecular dynamics simulator to simulate a nano-ECAP environment. Four single-crystal magnesium samples with different initial orientations were processed. The samples had dimensions of 20 nm × 20 nm×50 nm Figure 1 shows the same MD setup used in a previous study [16]. The die has a channel angle (Φ) of 90° and an AOB angle (Ψ) of 20°, which is also referred to as the deformation zone. The shear plane is OC and the shear direction is parallel to OC. The die was set as a rigid body and made one atom thick to minimize the atoms in the system and subsequently reduce the computation time. A cross-sectional area of 21 nm × 21 nm was used for the die to allow for expansion during equilibration and relaxation and to minimize grinding during compression. The four samples were labeled according to their initial orientations. The X-Basal sample was oriented in such a way that the basal plane {0001} was parallel to the x-axis/ED, and the X-Prism sample had its prismatic plane $\left\{10\bar{1}0\right\}$ oriented at 60° with the x-axis/ED, as shown in Figure 1. The S-basal sample was oriented in such a way that the basal plane was parallel to the shear plane, and the S-Prism sample had its prismatic plane at a 75° angle with the x-axis/ED, as shown in Figure 1.

Before ECAP, equilibration was done at 300 K for 40 ps. A timestep of 0.002 ps and the canonical (NVT) ensemble was used throughout the simulation, where *N* is the total number of atoms in the system, *V* is the volume, and *T* is the temperature. The NVT ensemble was used because the pressure is a variable during ECAP, and constant temperature is required to simulate cold-ECAP. A temperature of 300 K was maintained using a Nosé–Hoover thermostat. An infinite mass piston was used for compression at a rate of 2.0 Å/ps. The samples were processed by ECAP for 254 ps and then relaxed for 46 ps for a total of 300 ps. The interatomic potential was computed using the modified embedded atom model (MEAM) potential developed by Kim et al. [17]. The potential was used for its ability to reproduce the basic equilibrium properties of magnesium, namely, elastic constants, phonon-dispersion curves, vacancy formation, migration energy, stacking fault energy, and surface energy. Therefore, the MEAM potential is suitable for the current study. After the run, Open Visualization Tool (OVITO) software [18]was used to analyze the data and polyhedral template matching (PTM) algorithm [19]was used to analyze the local crystalline structure, misorientation angle, and grain structure. Furthermore, dislocation and atomic strain analyses were performed.

## 3 Results

### 3.1 Slip and dislocation

**Figure 2:**
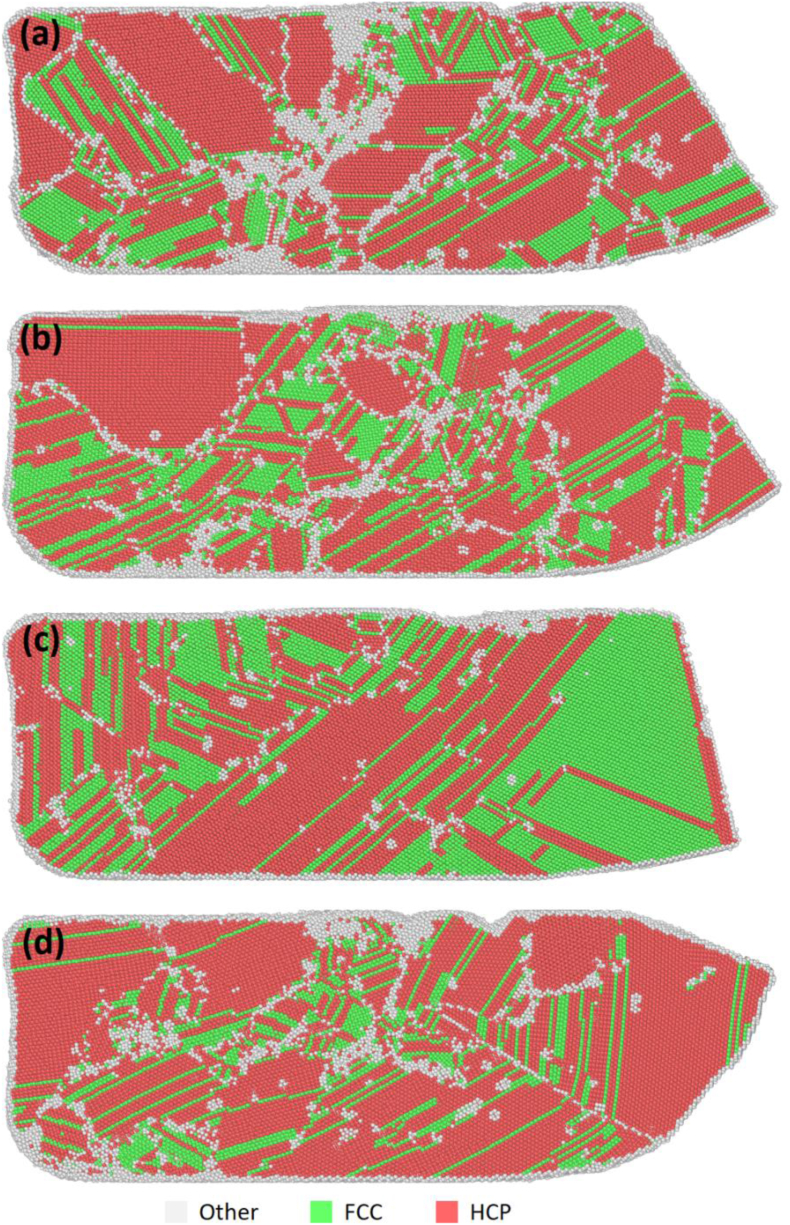
The crystal structure of the samples after ECAP (FCC atoms are green, HCP atoms are red, and random/amorphous atoms are white); (a) X-Basal; (b) X-Prism; (c) S-Basal; (d) S-Prism (refer to PTM supplementary videos 1-4for the full deformation process).

**Figure 3:**
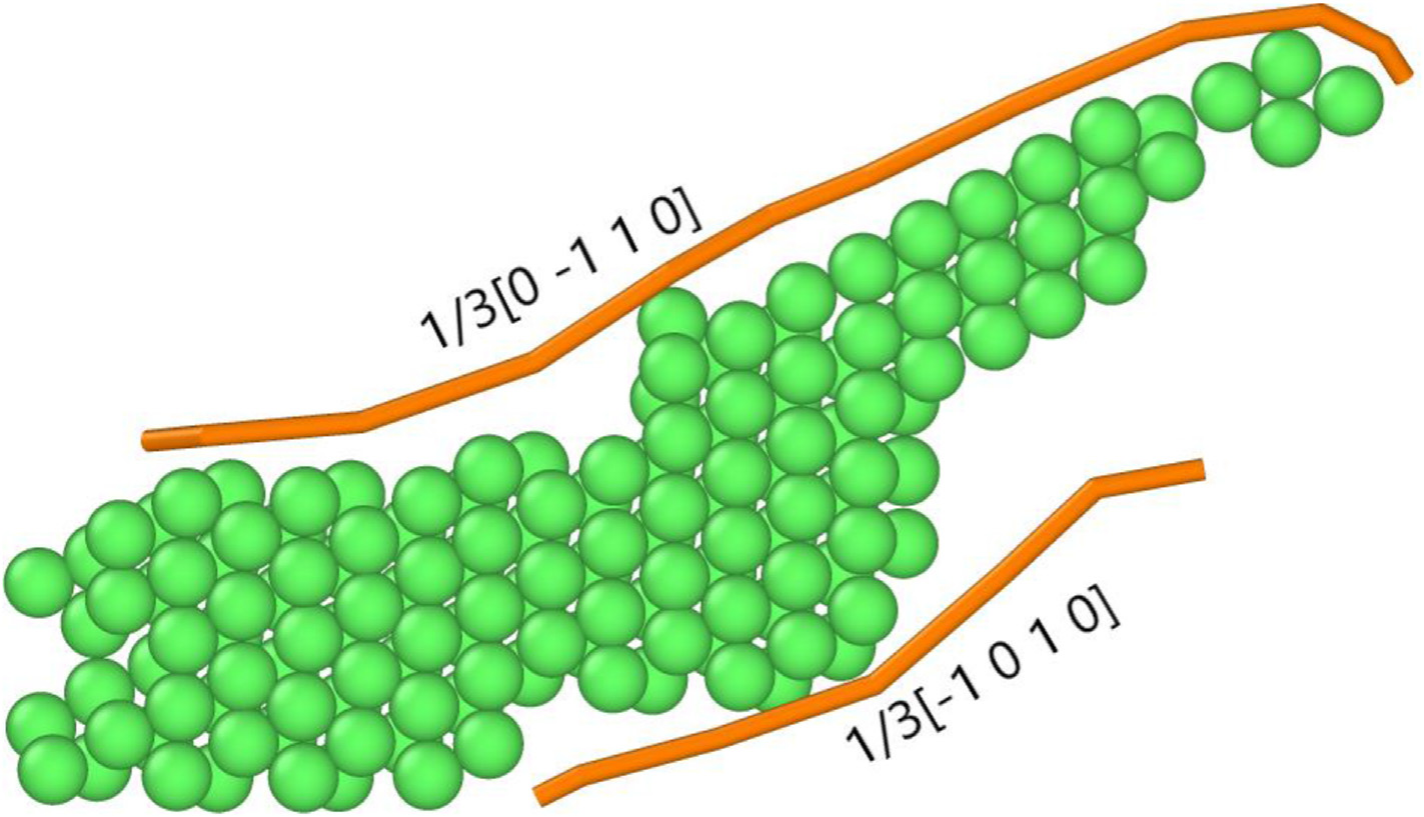
Stacking fault accompanied by a leading and trailing 1/3 < $1\bar{1}00$ > dislocation.

Table 1 shows the percentages of HCP, face-center cubic (FCC), and random atoms present after one-pass ECAP. The percentage of the FCC structure indicates the amount of stacking faults present in the sample. The percentage of random atoms indicates the percentage of atoms with an amorphous structure, such as grain boundaries and point defects. Although most of the samples showed similar trends, the S-Basal sample stood out with its high FCC structure content, as shown in Table 1. Figure 2(a-d) shows the crystal structure of all the samples after ECAP. The green atoms represent the stacking faults/FCC structure, the red atoms represent the HCP structure, and the white atoms represent the random/amorphous structure. A slip on the basal plane in the < $1\bar{1}00$ > direction was observed in all the samples, as shown in Figure 3. This slip was always accompanied by two 1/3 < $1\bar{1}00$ > partial or irregular dislocations with Burger's vector close to 1/3 < $1\bar{1}00$ >, forming a leading and trailing dislocation as shown in Figure 3. The slip resulted in the formation of stacking faults and changed the structure of the slipped plane from HCP to FCC.

**Table 1:** The percentage of HCP, FCC, and random structure present after ECAP.

Sample	HCP	FCC	Random
X-Basal	55.7%	21.9%	23.4%
X-Prism	51.1%	20.8%	29.1%
S-Basal	48.5%	35.8%	15.7%
S-Prism	62.1%	14.9%	24.4%

**Figure 4:**
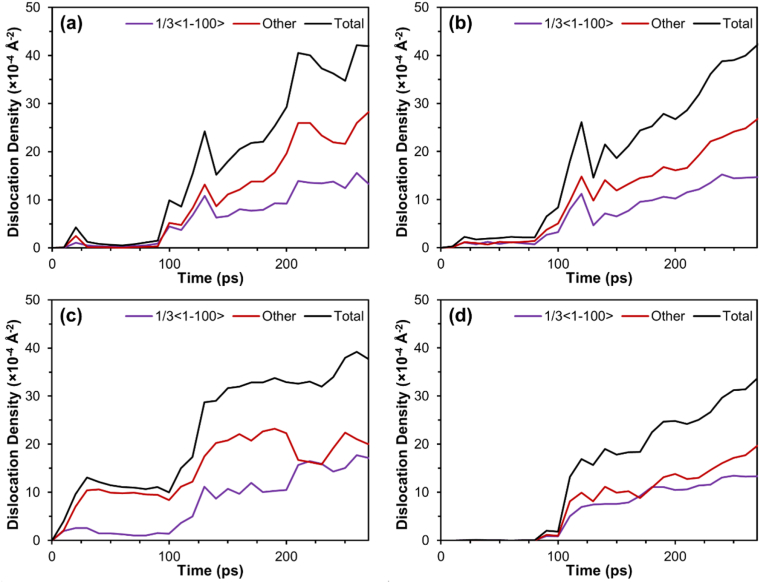
Dislocation density for each sample (a) X-Basal; (b) X-Prism; (c) S-Basal; (d) S-Prism.

As indicated in Figure 4(a-d), changes in the dislocation density of the $1/3<1\bar{1}00>$ partial dislocation, “other dislocation”, and total dislocations were observed. “Other dislocation” refers to dislocations with an irregular Burger's vector, and the $1/3<1\bar{1}00>$ dislocation is formed during stacking faults. In all the samples, the $1/3<1\bar{1}00>$ dislocation and “other dislocation” accounted for most of the dislocations present while exhibiting the same trend, but “other dislocation” is higher than the $1/3<1\bar{1}00>$ dislocation.

The ECAP process starts at 10ps, and the dislocations seen prior to that is due to the sample hitting the die. The X-Prism sample Figure 4(b) had the highest dislocation density. It initially exhibited a spike in dislocations corresponding to the start of extrusion, followed by a steady increase in dislocation density. The X-Basal sample Figure 4(a) came in second place, exhibiting a similar trend of a sharp spike in dislocation dislocations at the start of extrusion, followed by a steady increase in dislocation density then only a small change in dislocation density toward the end. Additionally, a spike in dislocations can be observed at around 12 ps, this corresponds with the formation of stacking faults. As shown in Figure 4(d), the S-Prism sample initially experienced a sharp rise followed by a steady increase in dislocation density throughout the process. However, it still had fewer dislocations than the previous samples. The S-Basal sample Figure 4(c) experienced dislocation from the beginning, where the dislocation increased sharply and then remained constant; the sample experienced another sharp rise, followed by another plateau. The sample had the least amount of dislocations compared with the other samples.

### 3.2 Twinning

**Figure 5:**
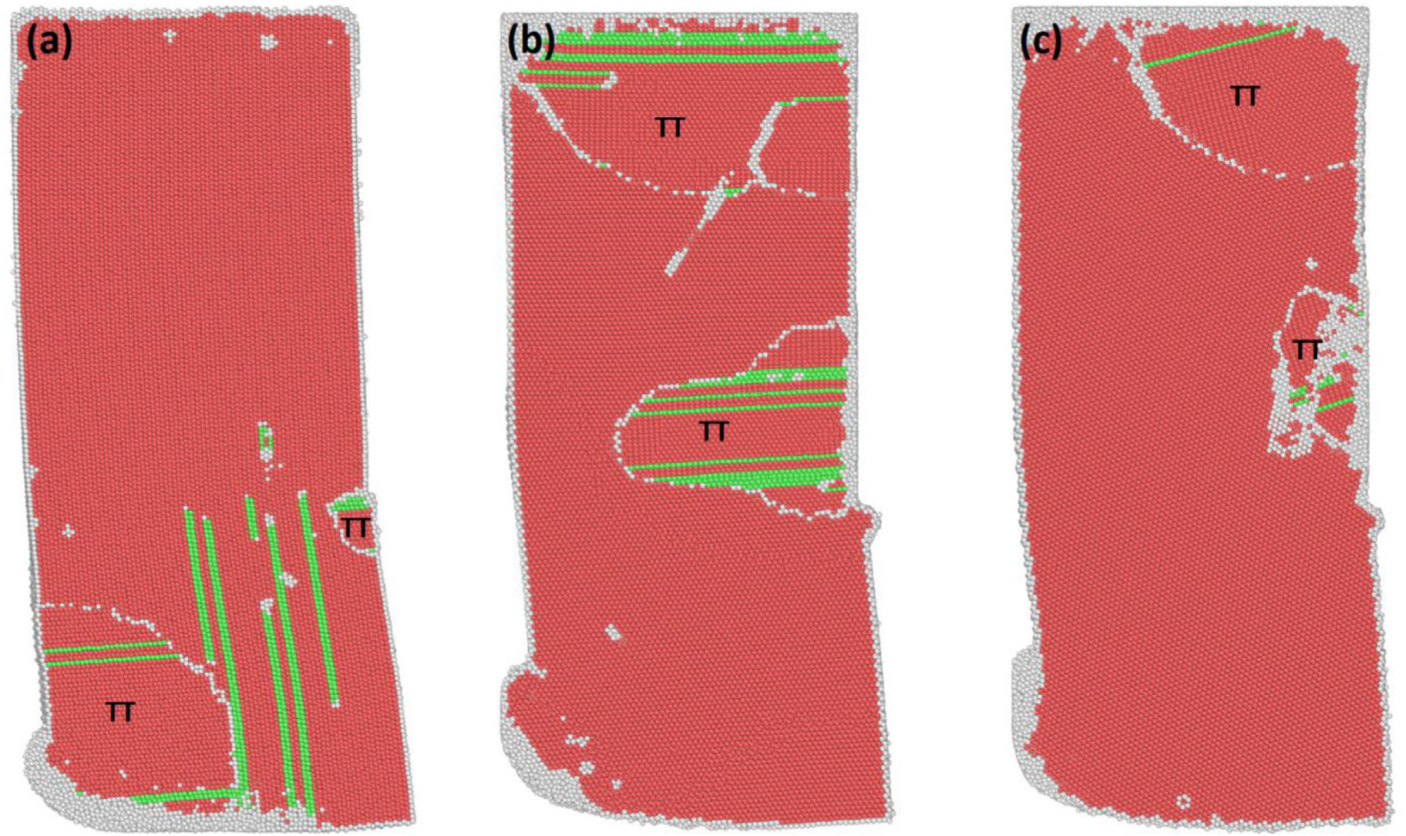
The formation of $\left\{10\bar{1}2\right\}$ tension twin upon the start of compression at 110 ps (time step 55000) in; (a) X-Basal; (b) X-Prism; (c) S-Prism.

**Figure 6:**
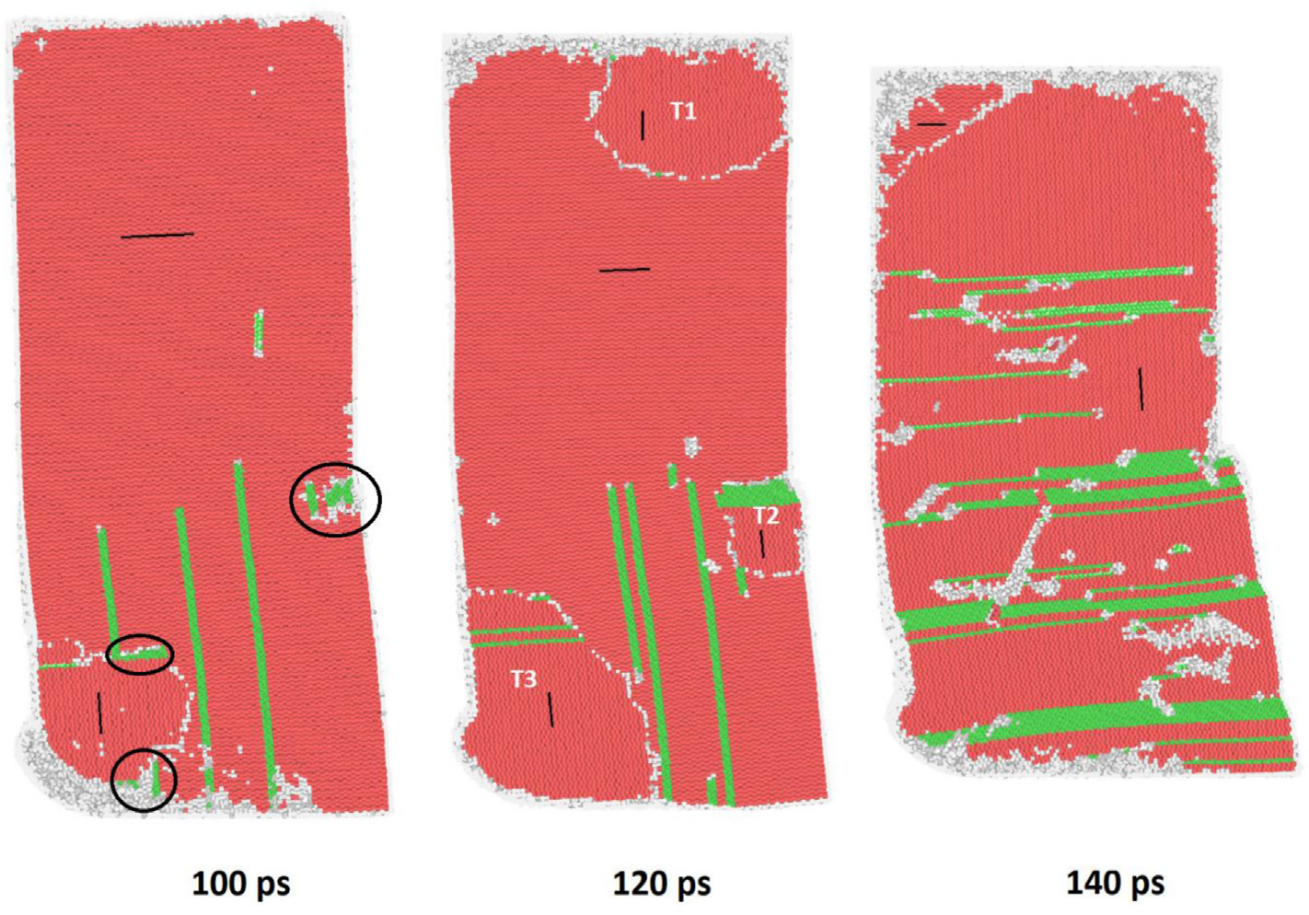
Crystal structure of X-Basal sample during ECAP with black lines representing the c-axis illustrating the formation of stacking faults in the black circles at 100 ps (time step 50000) followed by the nucleation $\{10\bar{1}2\}$ tension twin in T1, T2, and T3 at 120ps (time step 60000) spreading to cover the entire crystal at 140 ps (time step 70000). (refer to supplementary video PTM-XB for the full process).

Twinning was observed in all the samples, with the $\left\{10\bar{1}2\right\}$ tension twin being the most common because it is the most common twin in magnesium [14]. The $\left\{10\bar{1}2\right\}$ tension twin developed during the compression phase of ECAP and nucleated in high-stress regions, such as the curved part of the die or the piston. Figure 5(a-c) illustrates the nucleation of the $\left\{10\bar{1}2\right\}$ tension twins, and Figure 6 shows how T1, T2, and T3 grew at 120 ps and merged to cover the entire sample. Initially, T3 had already nucleated at 100 ps, and stacking faults were observed at the leading front which helped the twin grow even further. Meanwhile, T2 had not yet nucleated, and a concentration of stacking faults was observed at the exact location. The spike in dislocation at around 12 ps in Figure 4(a) and **(b)** correspond with the formation of stacking faults and the nucleation of the $\left\{10\bar{1}2\right\}$ tension twins. The stacking faults played a crucial role in the formation of the $\left\{10\bar{1}2\right\}$ tension twin, further evidence is provided in the PTM videos. The twinning effectively changed the orientation of the entire sample, as shown by the black lines, which indicate the c-axis direction in the HCP structure.

A misorientation angle of 86° was measured across the T1, T2, and T3 twin boundaries, which is characteristic of the $\left\{10\bar{1}2\right\}$ tension twin. Similarly, the nucleation of the $\left\{10\bar{1}2\right\}$ tension twin during compression was also observed in the X-Prism and S-Prism samples, as illustrated in the supplementary videos of PTM-XP and PTM-SP. In contrast, multiple twins of different types were observed after the ECAP process. The $\left\{11\bar{2}1\right\}$, $\left\{10\bar{1}1\right\}$, and $\left\{11\bar{2}2\right\}$ twins were also observed in all the samples, except for the S-Basal sample due to its orientation.

### 3.3 Strain and deformation

**Figure 7:**
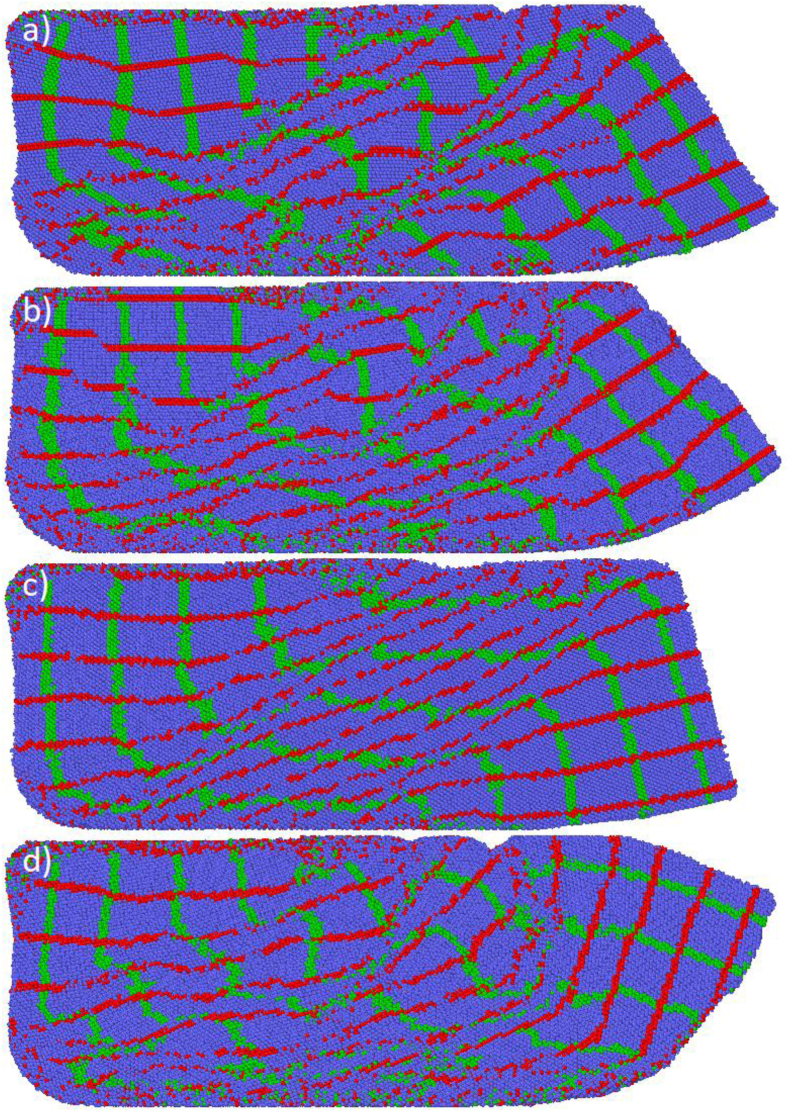
The deformation pattern after ECAP; (a) X-Basal; (b) X-Prism; (c) S-Basal; (d) S-Prism (refer to deformation supplementary videos 5-8for the full process).

Figure 7(a-d) shows the deformation patterns of all samples after ECAP. The samples were initially marked with vertical (green) and horizontal (red) lines to observe the deformation behavior. Straight, uniform, and equidistant lines at 45° were expected to exist in all the samples after ECAP. However, only the S-Basal sample in Figure 7(c) exhibited such a pattern. The X-Basal and X-Prism samples exhibited similar patterns, with the red lines forming a straight diagonal pattern and the green lines forming waves. By contrast, the deformation behavior of the S-Prism sample was entirely chaotic, with the red lines remaining almost horizontal and mixing with the green lines near the bottom. Green waves were also formed at the center of the sample.

The chaotic nature of the red and green lines indicated deformation severity during ECAP, as confirmed by the strain values in Table 2. The sample's atomistic shear and volumetric strain were calculated using the Green–Lagrangian strain tensor in OVITO, and the average strains across the samples are shown in Table 2. The S-Basal sample with the most organized and uniform lines had the lowest shear and volumetric strain, and the S-Prism sample with the most chaotic pattern had the highest shear and volumetric strain. The regions where the lines were entangled and the green lines that formed valleys in Figure 7 coincided with the region possessing high strain and grain fragmentation.

**Table 2:** The volumetric and shear strain for each sample after ECAP.

Strain	X-Basal	X-Prism	S-Basal	S-Prism
Shear Strain	2.05	1.96	1.63	2.22
Volume Strain	1.07	1.02	0.82	1.18

**Figure 8:**
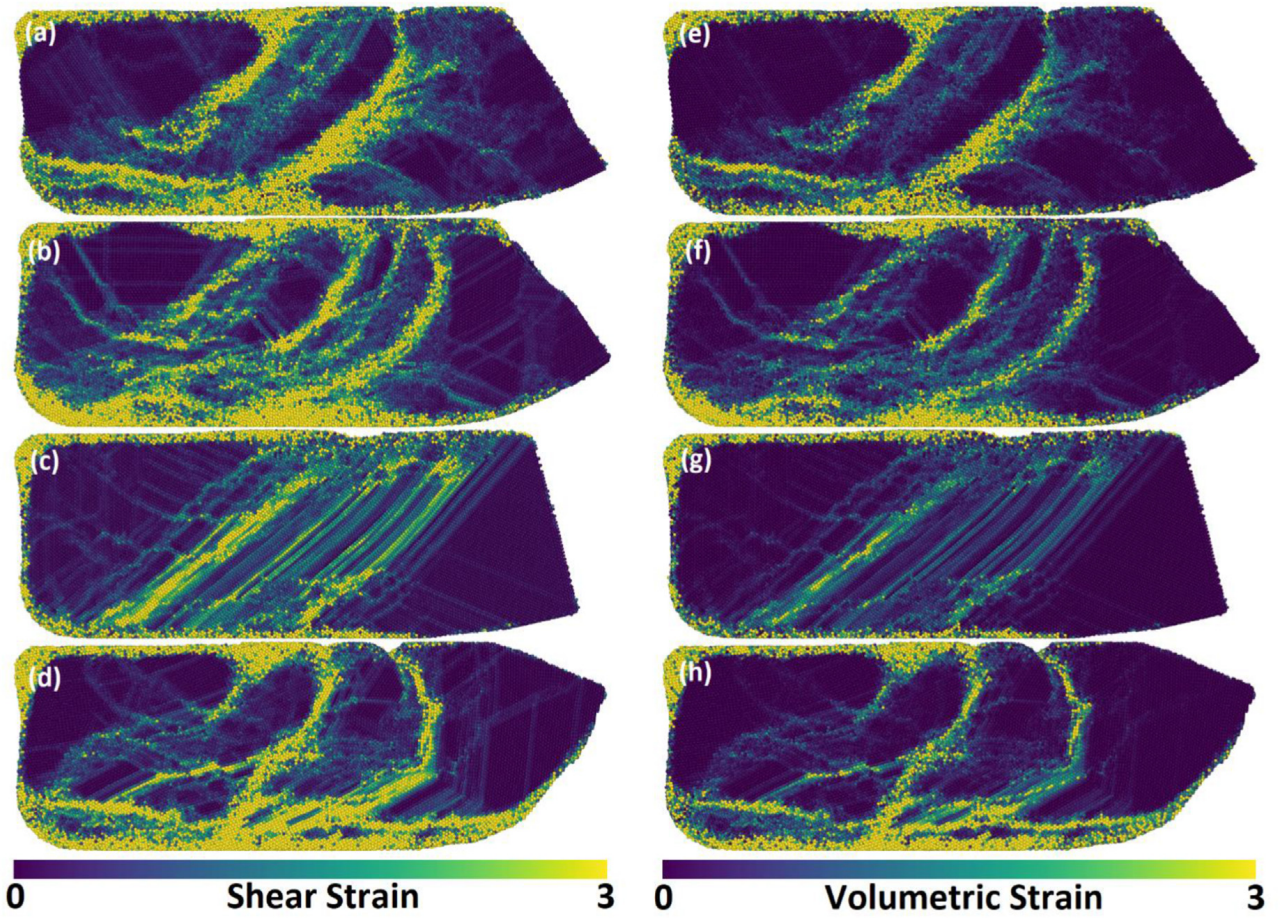
The shear and volumetric strains for each sample after ECAP; (a) X-Basal; (b) X-Prism; (c) S-Basal; (d) S-Prism; (e) X-Basal; (f) X-Prism; (g) S-Basal; (h) S-Prism.

Figure 8(a-h) presents the shear and volumetric strain concentration after ECAP. The strain and deformation patterns fit perfectly with the entangled lines. Volumetric strain occurred in the same location where shear did at a high intensity. The strain line formed at approximately 45° with the x-axis/ED. Meanwhile, the strain was concentrated in a few locations in the X-Basal, X-Prism, and S-Prism samples. The S-Basal sample had equally distributed strain across the impacted region, which explains why the deformation lines are arranged in an orderly manner in Figure 8(c).

## 4 Discussion

### 4.1 Resolved shear stress

ECAP involves two modes of deformation: compression, which occurs during the initial stages of ECAP, and shear, which occurs after extrusion begins. Accordingly, the resolved shear stress ($\tau_{RSS}$) shown in Eq. (1) is the shear component (m) of the stress applied to the slip system. $\tau_{RSS}$ identifies the most active slip system during the deformation stages of ECAP, as reported in a previous study [16]. The Schmid and shear factors are shear components. The Schmid factor is associated with tensile and compressive stresses and ranges from 0 to 0.5. By contrast, the shear factor is associated with the shear stresses found in the deformation zone and ranges from 0 to 1. The Schmid and shear factors for each slip system in this study were calculated using Eq. (2).

(1) $$\tau_{RSS}=\frac{F}{A}\;m$$

and,

(2) m=cos(α)cos(β)

where F/A is the applied stress, m is the shear component, and α is the angle between the compressive force and slip plane for the Schmid factor and the angle between the shear plane and slip plane for the shear factor. Meanwhile, β is the angle between the compressive force and slip direction for the Schmid factor and the angle between the shear force and the slip direction for the shear factor.

Table 3 presents the component with the highest value for each plane family. The high Schmid factor indicates that during the compression phase of ECAP, the prismatic $\left\{10\bar{1}0\right\}<11\bar{2}0>$ slip system was the most active in all the samples, except for the S-Basal sample where the basal $\left\{0001\right\}<11\bar{2}0>$ was the dominant slip system. This result partially explains why the 1/3 $<1\bar{1}00>$ dislocation was very prominent in the S-Basal sample, as shown in Figure 4(c), because the 1/3 $<1\bar{1}00>$ partial dislocation is generally formed through the dissociation of the $\left\{0001\right\}<11\bar{2}0>$ dislocation [20], as shown in Eq. (3).

(3) $$\frac{1}{3}\left[11\bar{2}0\right]\to \frac{1}{3}\left[10\bar{1}0\right]+\frac{1}{3}\left[01\bar{1}0\right]$$

**Table 3:** The Schmid and Shear factor of the most active slip systems in each slip plane for each sample during the compression and shear phase of ECAP with bold indicating the most active.

Slip System	Schmid Factor				Shear Factor			
	X-Basal	X-Prism	S-Basal	S-Prism	X-Basal	X-Prism	S-Basal	S-Prism
Basal $\left\{0001\right\}<11\bar{2}0>$	0	0	**0.433**	0	0.500	0	**0.866**	0
Prismatic $\left\{10\bar{1}0\right\}<11\bar{2}0>$	**0.433**	**0.433**	0.217	**0.500**	0.217	**0.933**	0	**0.750**
Pyramidal I $\left\{10\bar{1}1\right\}<11\bar{2}\bar{3}>$	0.401	0.401	0.388	0.431	**0.915**	0.727	0.390	0.632
Pyramidal II $\left\{11\bar{2}2\right\}<11\bar{2}\bar{3}>$	0.335	0.335	0.250	0.416	0.708	0.513	0.434	0.376

### 4.2 Twin deformation

Twins were formed early during compression before the extrusion process started in all the samples, except for the S-Basal sample, as presented in Figure 5(a-c) and the supplementary PTM videos. Notably, the $\left\{10\bar{1}2\right\}$ tension twin is the most common twin in magnesium [14]. It occurs in high-stress regions, such as the region pressed by the piston (T1), at the bottom where the sample contacts the die (T3), or at the edge where both channels meet (T2) as illustrated in Figure 6. For HCP materials with $c/a<\sqrt{3}$ such as magnesium, the $\left\{10\bar{1}2\right\}$ tension twin occurs when compression is applied parallel to the {0001} basal plane or when tension is applied parallel to the $\left\{10\bar{1}0\right\}$ prismatic plane [10]. For the X-Basal, X-Prism, and S-Prism samples in the current study, compression was applied parallel to the {0001} basal plane. Further verification is provided in Table 3 by the Schmid factor calculation, where a value of 0 was obtained, indicating that the compression force was parallel to the basal plane. Additionally, stacking faults were observed at the location of twin nucleation prior to twin formation. *Zhang et al.* [14] reported that stacking faults are a precursor for the $\{10\bar{1}2\}\;<10\bar{1}1>$ tension twin, which was also observed in our samples as seen in Figure 6. In brief, the mechanism for the $\left\{10\bar{1}2\right\}$ tension twin formation is a series of stacking faults occurring in different directions, where an initial stacking fault changes the crystal structure from HCP to FCC, followed by another stacking fault in a different direction changing the crystal structure back to HCP. This crystal structure change results in a twin boundary with a mirror at 86°.

During compression, the $\left\{10\bar{1}2\right\}$ tension twin nucleated in the X-Basal, X-Prism, and S-Prism samples and grew to cover the entire sample, as illustrated in Figure 6. The formation and growth of the twin effectively changed the orientation of the sample. Initially, the c-axis [0001] of the sample pointed toward the x-axis/ED, but after the nucleation of the $\left\{10\bar{1}2\right\}$ tension twin, the c-axis direction changed to point towards the y-axis/insert direction (ID). Consequently, a modified shear factor was calculated and is shown in Table 4 in consideration of the orientation change caused by twining. By contrast, the S-Basal sample did not nucleate a $\left\{10\bar{1}2\right\}$ tension twin due to its unique orientation. Meanwhile, the $\left\{10\bar{1}3\right\}$ twin was observed momentarily during compression and disappeared shortly after the start of the extrusion phase, as shown in the supplementary video of PTM-SB.

**Table 4:** Modified Shear factor calculation after the nucleation of the $\left\{10\bar{1}2\right\}$ tension twin.

Slip System	Shear Factor			
	X-Basal	X-Prism	S-Basal∗	S-Prism
Basal $\left\{0001\right\}<11\bar{2}0>$	0.433	0.500	**0.866**	0.750
Prismatic $\left\{10\bar{1}0\right\}<11\bar{2}0>$	0.217	0.217	0	0.188
Pyramidal I $\left\{10\bar{1}1\right\}<11\bar{2}\bar{3}>$	**0.883**	0.849	0.390	0.694
Pyramidal II $\left\{11\bar{2}2\right\}<11\bar{2}\bar{3}>$	0.823	**0.946**	0.434	**0.774**

∗ S-Basal sample did not change because twinning did not occur during compression.

### 4.3 Stacking faults

Magnesium possesses a hexagonal closed-pack crystal structure. The {0001} basal plane is arranged in an alternating …ABABAB... stacking sequence in a perfect crystal. Given that the basal plane is the densest, it is the preferred slip system during deformation, which forms stacking faults, with the intrinsic **I**_**1**_ and **I**_**2**_ and the extrinsic **E** stacking faults being the most common [21]. **I**_**1**_ is a single-plane stacking fault formed by removing a basal plane, followed by a shear displacing the remaining planes by $1/3<10\bar{1}0>$. **I**_**2**_ is a two-plane stacking fault formed by directly shearing the lattice by a $1/3<10\bar{1}0>$ displacement. **E** is a three-plane stacking fault formed by the insertion of an extra basal plane [21, 22].

When a stacking fault is formed, the faulted plane changes its structure from HCP to FCC. Consequently, the percentage of the FCC structure formed after ECAP can be used to measure the stacking fault frequency. As indicated in Table 1, the S-Basal sample had the highest percentage of the FCC structure at 35.8%, and the X-Basal and X-Prism samples had a similar FCC content of roughly 21%. The S-Prism sample had the lowest FCC content at 15%, but a high modified shear factor as shown in Table 4. This discrepancy can be explained by the high dislocation recovery when compared to the other samples, in the PTM supplementary videos, it is observed that a considerable portion of FCC/stacking faults formed revert to the normal HCP structure after leaving the deformation zone. In contrast, the reason for the high FCC content in the S-Basal sample is its unique and unchanged orientation during ECAP. The basal plane is oriented such that it is parallel to the shear plane, with the $\left[10\bar{1}0\right]$ direction being parallel to the shear direction, thus taking full advantage of the basal stacking fault mechanism directly, bypassing the dissociation of the $1/3\left[11\bar{2}0\right]$ dislocation in Eq. (3), and directly slipping by $1/3<10\bar{1}0>$. The uniform deformation pattern in the S-Basal sample, which is shown in Figure 8(c), can be explained by the relation between the shear and basal planes in the S-Basal sample.

### 4.4 Strain

In metals, stacking faults are used as a mechanism to accommodate strain; thus, samples with many stacking faults experience low strain. Accordingly, comparing the percentage of stacking faults in Table 1 and the shear strain in Table 2, a relationship between strain and the abundance of stacking faults can be observed. Given that the S-Basal sample takes advantage of the basal stacking fault mechanism, it had the most stacking faults and the lowest shear strain at 1.63. Meanwhile, the S-Prism sample had the fewest stacking faults and the highest shear strain of 2.22. Furthermore, when the 0.833 shear factor of the S-Basal sample is considered for the basal slip system, it becomes clear why the S-Basal sample has the lowest strain. The $\left\{0001\right\}<11\bar{2}0>$ basal is the preferred slip system for the HCP crystal structure; the critical resolved shear stress of these non-basal slip systems at room temperature is approximately 100 times that of the basal slip system [14, 21].

When the modified shear factor in Table 4 is considered, the reason behind the different strain values becomes increasingly apparent. After the nucleation of the $\left\{10\bar{1}2\right\}$ tension twin, the X-Basal and X-Prism samples ended with a very similar orientation: the c-axis pointed toward the y-axis/ID, and the only difference was that the samples were rotated by 30° around the y-axis/ID apart from each other. Consequently, both samples ended with similar deformation and shear patterns, as illustrated in Figures 7(a,b) and **8(a,b)**. Additionally, the modified shear factor for both samples was very high, that is, 0.883 for the Pyramidal I slip system in the X-Basal sample and 0.946 for the Pyramidal II slip system in the X-Prism sample. The application of the modified shear factor can also explain why both samples experienced a similar magnitude of strain. By contrast, the S-Prism sample had the lowest shear factor of 0.774 of a difficult-to-activate slip system compared with the other samples.

(4) $$\gamma =2\;\cot\left(\frac{\Phi +\Psi }{2}\right)+\Psi \;\csc\left(\frac{\Phi +\Psi }{2}\right)$$

(5) ε=γ3

The theoretical shear and volumetric strain obtained after ECAP are 1.83 and 1.06, respectively, as calculated with Eqs. (4) and (5) [23]. The discrepancy observed between the theoretical and actual values shown in Table 2 can be attributed to two factors. First, Eq. (4) considers simple shear to be the mode of deformation during ECAP, with the entire deformation process occurring in the deformation zone; it does not consider the shear strain due to normal stress in the compression phase of ECAP. Second, it considers the deformation to be homogenous and isotropic, and it does not take into account the initial grain orientation and the anisotropic nature of magnesium. For anisotropic materials, such as magnesium, different deformation behaviors are exhibited depending on the initial orientation of the grain, especially because <c+a> deformation requires 100 times more strain compared with <a> deformation, as shown in Table 4.

Additionally, *Ventura et al.* [24] reported similar results for the compression of magnesium single crystal micro-pillars with similar orientations. Compression tests were performed under different strain rates ranging from 10^−4^ s^−1^ to 500 s^−1^. It was observed that twinning increased as the strain rate increased, and at high strain rates the twin would grow to cover the pillar completely. Furthermore, the deformation behavior observed by *Ventura et al* was identical to what occurs during the compression phase.

In summary, the deformation in single-crystal magnesium during ECAP can be divided into two stages: deformation due to compression and deformation due to shear. Initially, compression is the dominant mode of deformation, during which stacking faults are created and act as a precursor to the $\left\{10\bar{1}2\right\}$ tension twin. When the sample enters the deformation zone, simple shear becomes the dominant deformation; it shears the sample as it passes through. At this time, the majority of stacking faults are introduced. The strain starts to accumulate, which introduces dislocations and defects until the strain is high enough to fragment the crystal. Additionally, the results of this study are in line with the results obtained experimentally by *Kitahara et al.* [25]. Similarly, they processed samples of single-crystal magnesium with different initial orientations at room temperature by using a die with a 90° channel angle (Φ). Finally, understanding the deformation behavior of single crystals will provide a better understanding of the mechanism behind polycrystal deformation and grain fragmentation. However, further works on polycrystal deformation is required.

## 5 Conclusion

In conclusion, molecular dynamics was used to investigate the ECAP of single-crystal magnesium with different initial orientations. The following points were observed.

1. The initial orientation played a substantial role in the strain and deformation experienced by the samples during both stages of deformation. The S-Prism sample had the best performance due to the high shear and volumetric strain of 2.22 and 1.18, respectively.
2. During ECAP, the deformation could be divided into two stages; compressions that occurred initially before extrusion began and simple shear that occurred in the deformation zone during extrusion.
3. The X-Basal, X-Prism, and S-Prism samples nucleated the $\left\{10\bar{1}2\right\}$ tension twin at the start of compression, and the $\left\{10\bar{1}2\right\}$ twin grew to immediately cover the entire sample, thereby effectively changing the orientation of the sample. The stacking faults acted as a precursor for twin formation; stacking faults were observed prior to the formation of the $\left\{10\bar{1}2\right\}$ tension twin and at the leading front of the twin boundary to promote further growth.
4. The S-Basal sample exhibited the lowest strain and the highest percentage of stacking faults. The basal plane was oriented parallel to the shear plane, and the shear direction was parallel to the $<10\bar{1}0>$ direction, taking full advantage of the basal stacking fault direction.
5. A correlation was observed between the shear factor and strain due to the calculation of the shear and Schmid factors. The high shear factor resulted in a low strain, as shown in the X-Basal, X-Prism, and S-Prism samples. Although the S-Basal sample had a similar shear factor, it was 100 times easier to activate the basal slip system compared with the pyramidal slip system in the other samples.
6. A discrepancy was noted between the theoretical and actual shear strain, and it was attributed to two factors. First, theoretical shear is considered to be simple shear occurring entirely in the deformation zone; it does not consider the shear strain due to the normal stress in the compression phase of ECAP. Second, deformation is considered to be homogenous and isotropic, and it does not take into account the initial grain orientation and the anisotropic nature of magnesium.

## Declarations

### Author contribution statement

Amro H. Altoyuri: Performed the experiments; Analyzed and interpreted the data; Contributed reagents, materials, analysis tools or data; Wrote the paper.

Junaidi Syarif: Conceived and designed the experiments; Analyzed and interpreted the data; Contributed reagents, materials, analysis tools or data; Wrote the papr

Zainuddin Sajuri: Analyzed and interpreted the data; Wrote the paper.

### Funding statement

This research did not receive any specific grant from funding agencies in the public, commercial, or not-for-profit sectors.

### Data availability statement

The raw/processed data required to reproduce these findings cannot be shared at this time as the data also forms part of an ongoing study.

### Declaration of interests statement

The authors declare no conflict of interest.

### Additional information

No additional information is available for this paper.

## Appendix A Supplementary data

mmc1 mmc1

mmc2 mmc2

mmc3 mmc3

mmc4 mmc4

mmc5 mmc5

mmc6 mmc6

mmc7 mmc7

mmc8 mmc8

## References

[1] Yang M., Liu Y.H., Liu J.A., Song Y.L. (2015). Effect of T6 heat treatment on corrosion resistance and mechanical properties of AM50 magnesium alloy. Mater. Res. Innovat..

[2] Ramalingam V.V., Ramasamy P., Das Kovukkal M., Myilsamy G. (2020). Research and development in magnesium alloys for industrial and biomedical applications: a review. Met. Mater. Int..

[3] Rahman M., Dutta N.K., Roy Choudhury N. (2020). Magnesium alloys with tunable interfaces as bone implant materials. Front. Bioeng. Biotechnol..

[4] Gopi K.R., Shivananda Nayaka H. (2017). Microstructure and mechanical properties of magnesium alloy processed by equal channel angular pressing (ECAP). Mater. Today Proc..

[5] Properties M., Silver A., Straumal B., Martynenko N., Temralieva D., Serebryany V., Tabachkova N., Shchetinin I., Anisimova N., Kiselevskiy M. (2020).

[6] Muralidhar A., Narendranath S., Shivananda Nayaka H. (2013). Effect of equal channel angular pressing on AZ31 wrought magnesium alloys. J. Magnes. Alloy..

[7] Kitahara H., Mayama T., Okumura K., Tadano Y., Tsushida M., Ando S. (2014). Anisotropic deformation induced by spherical indentation of pure Mg single crystals. Acta Mater..

[8] Ando S., Tsushida M., Kitahara H. (2010). Deformation behavior of magnesium single crystal in c-axis compression and a-axis tension. Mater. Sci. Forum.

[9] Tang Y., El-Awady J.A. (2014). Formation and slip of pyramidal dislocations in hexagonal close-packed magnesium single crystals. Acta Mater..

[10] Guo Y.F., Xu S., Tang X.Z., Wang Y.S., Yip S. (2014). Twinnability of hcp metals at the nanoscale. J. Appl. Phys..

[11] Wang S., Gong M., McCabe R.J., Capolungo L., Wang J., Tomé C.N. (2020). Characteristic boundaries associated with three-dimensional twins in hexagonal metals. Sci. Adv..

[12] Agarwal G., Dongare A.M. (2019). Deformation twinning in polycrystalline Mg microstructures at high strain rates at the atomic scales. Sci. Rep..

[13] Ostapovets A., Serra A. (2020). Review of non-classical features of deformation twinning in HCP metals and their description by disconnection mechanisms. Metals.

[14] Zhang Z., Peng J.H., Huang J., Guo P., Liu Z., Song S.C., Wang Y. (2020). {101¯2} twinning nucleation in magnesium assisted by alternative sweeping of partial dislocations via an intermediate precursor. J. Magnes. Alloy.

[15] Thompson A.P., Aktulga H.M., Berger R., Bolintineanu D.S., Brown W.M., Crozier P.S., in ’t Veld P.J., Kohlmeyer A., Moore S.G., Nguyen T.D., Shan R., Stevens M.J., Tranchida J., Trott C., Plimpton S.J. (2022). LAMMPS - a flexible simulation tool for particle-based materials modeling at the atomic, meso, and continuum scales. Comput. Phys. Commun..

[16] Syarif J., Altoyuri A., Mohamed I.F. (2022). Equal Channel angular pressing of single crystal aluminum: a molecular dynamics simulation. J. Mater. Res. Technol..

[17] Kim Y.M., Kim N.J., Lee B.J. (2009). Atomistic modeling of pure Mg and Mg-Al systems, calphad comput. Coupling Phase Diagrams Thermochem.

[18] Stukowski A. (2010). Visualization and analysis of atomistic simulation data with OVITO-the Open Visualization Tool. Model. Simulat. Mater. Sci. Eng..

[19] Larsen P.M., Schmidt Sø., Schiøtz J. (2016). Robust structural identification via polyhedral template matching. Model. Simulat. Mater. Sci. Eng..

[20] Wen L., Chen P., Tong Z.F., Tang B.Y., Peng L.M., Ding W.J. (2009). A systematic investigation of stacking faults in magnesium via first-principles calculation. Eur. Phys. J. B.

[21] Zhu S.Q., Ringer S.P. (2018). On the role of twinning and stacking faults on the crystal plasticity and grain refinement in magnesium alloys. Acta Mater..

[22] Wright A.F. (1997). Basal-plane stacking faults and polymorphism in AlN, GaN, and InN. J. Appl. Phys..

[23] Iwahashi Y., Wang J., Horita Z., Nemoto M., Langdon T.G. (1996). Principle of equal-channel angular pressing for the processing of ultra-fine grained materials. Scripta Mater..

[24] della Ventura N.M., Sharma A., Kalácska S., Jain M., Edwards T.E.J., Cayron C., Logé R., Michler J., Maeder X. (2022). Evolution of deformation twinning mechanisms in magnesium from low to high strain rates. Mater. Des..

[25] Kitahara H., Maruno F., Tsushida M., Ando S. (2014). Deformation behavior of Mg single crystals during a single ECAP pass at room temperature. Mater. Sci. Eng..

