# Supplementary figures and images for: Deformation behavior of single-crystal magnesium during Nano-ECAP simulation
Source: Heliyon. 2022 Nov 23;8(12):e11837. doi: 10.1016/j.heliyon.2022.e11837 (PMC9720040; doi:10.1016/j.heliyon.2022.e11837)

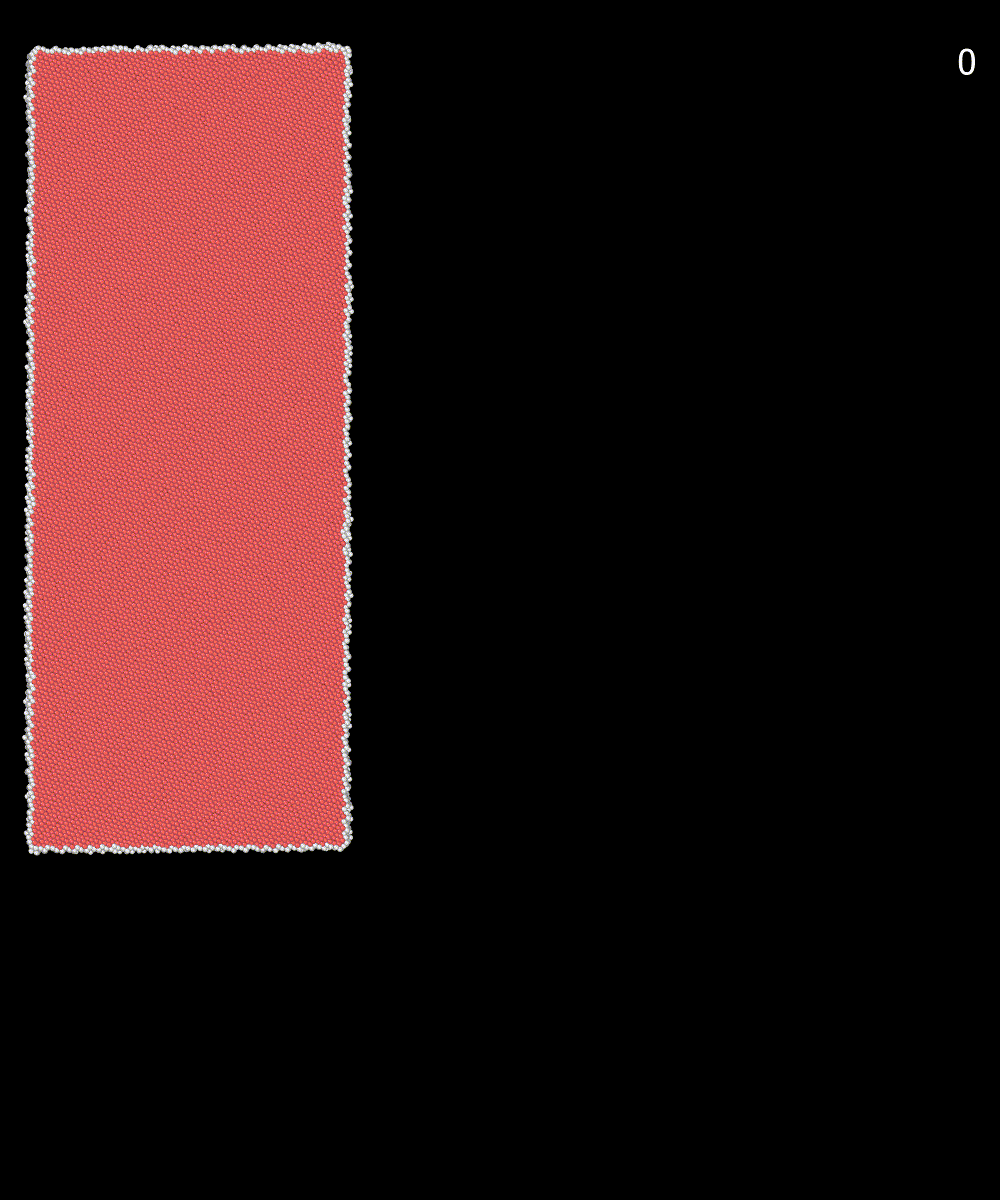

Supplement: mmc1 [file mmc1.gif]

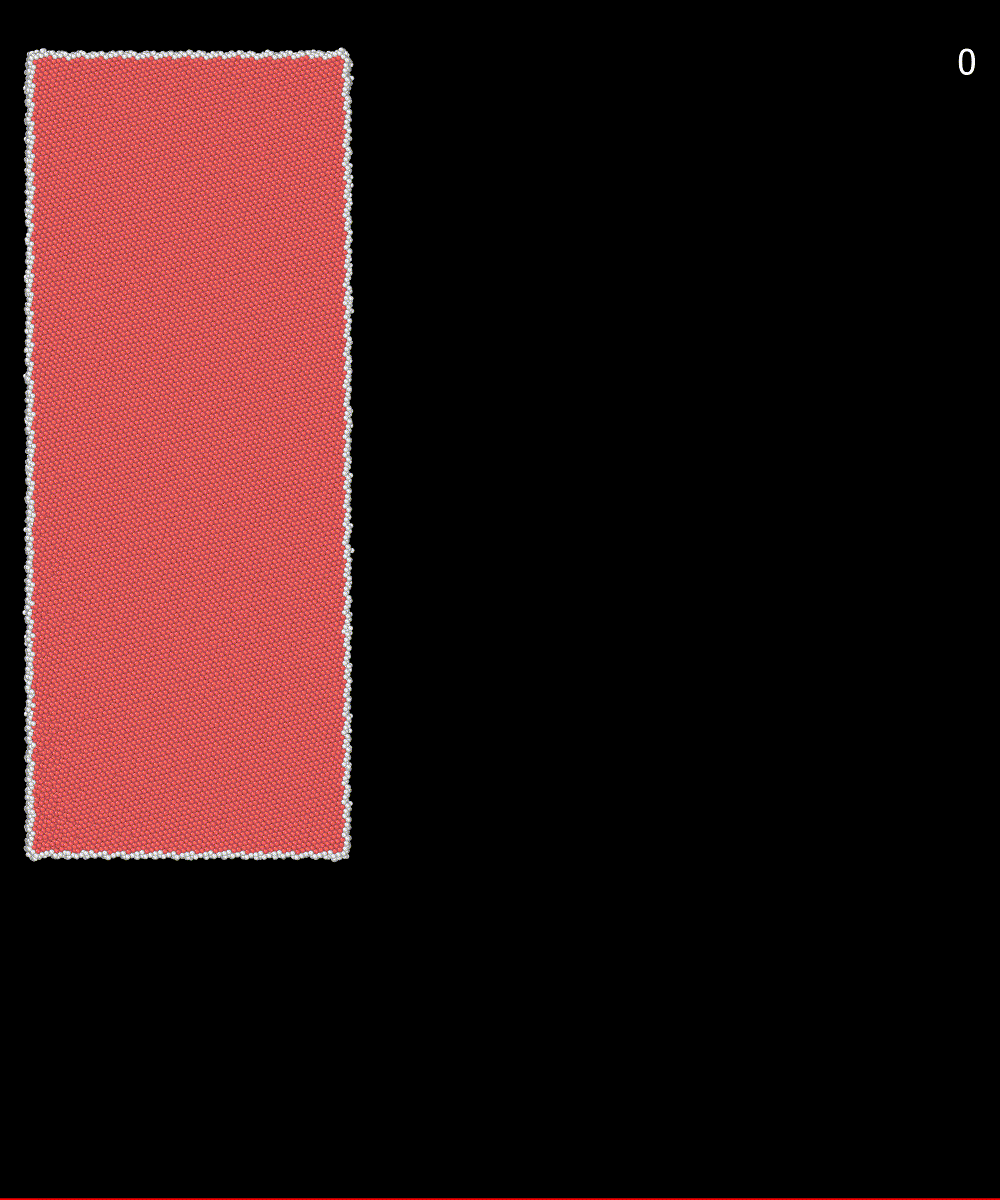

Supplement: mmc2 [file mmc2.gif]

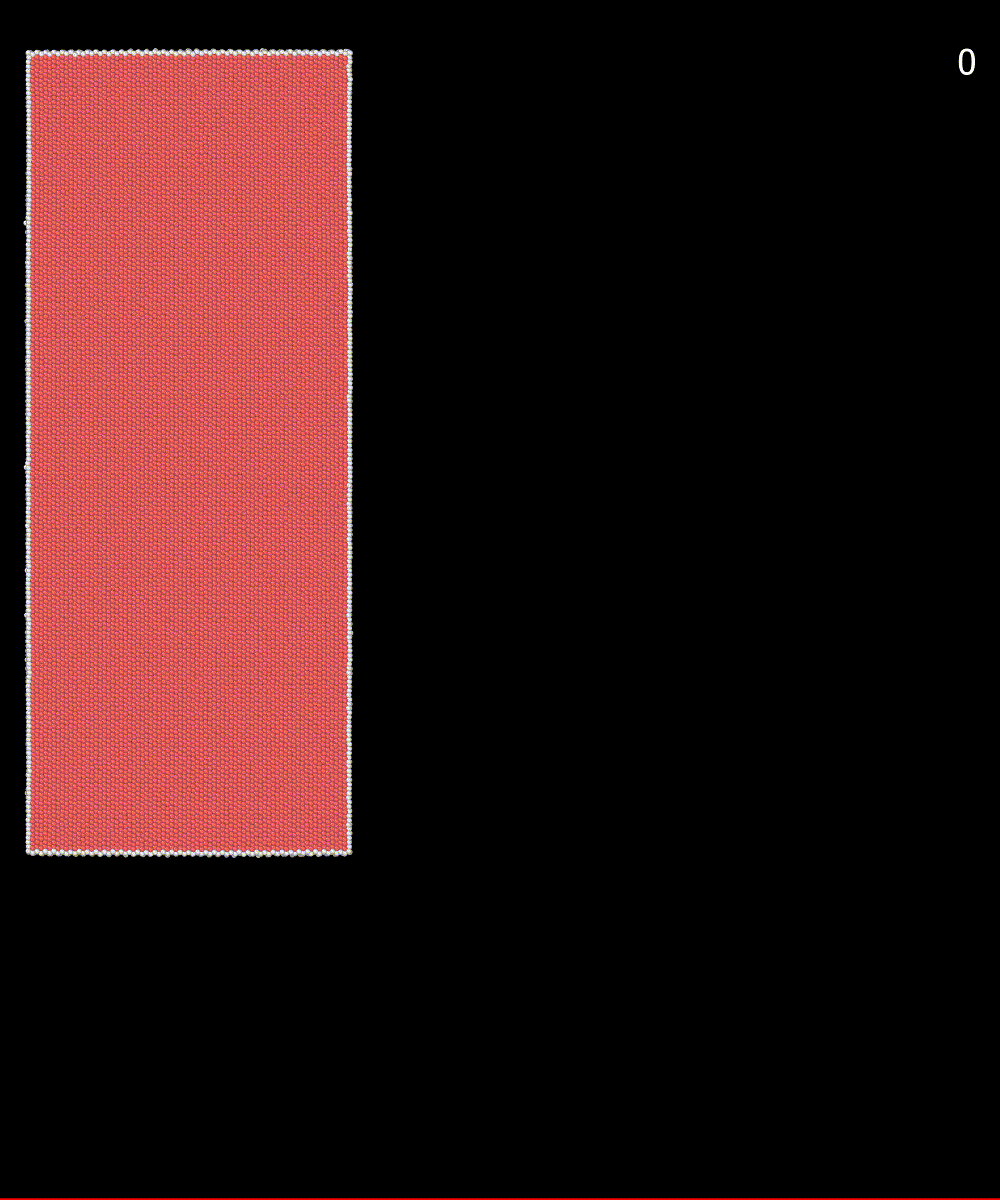

Supplement: mmc3 [file mmc3.gif]

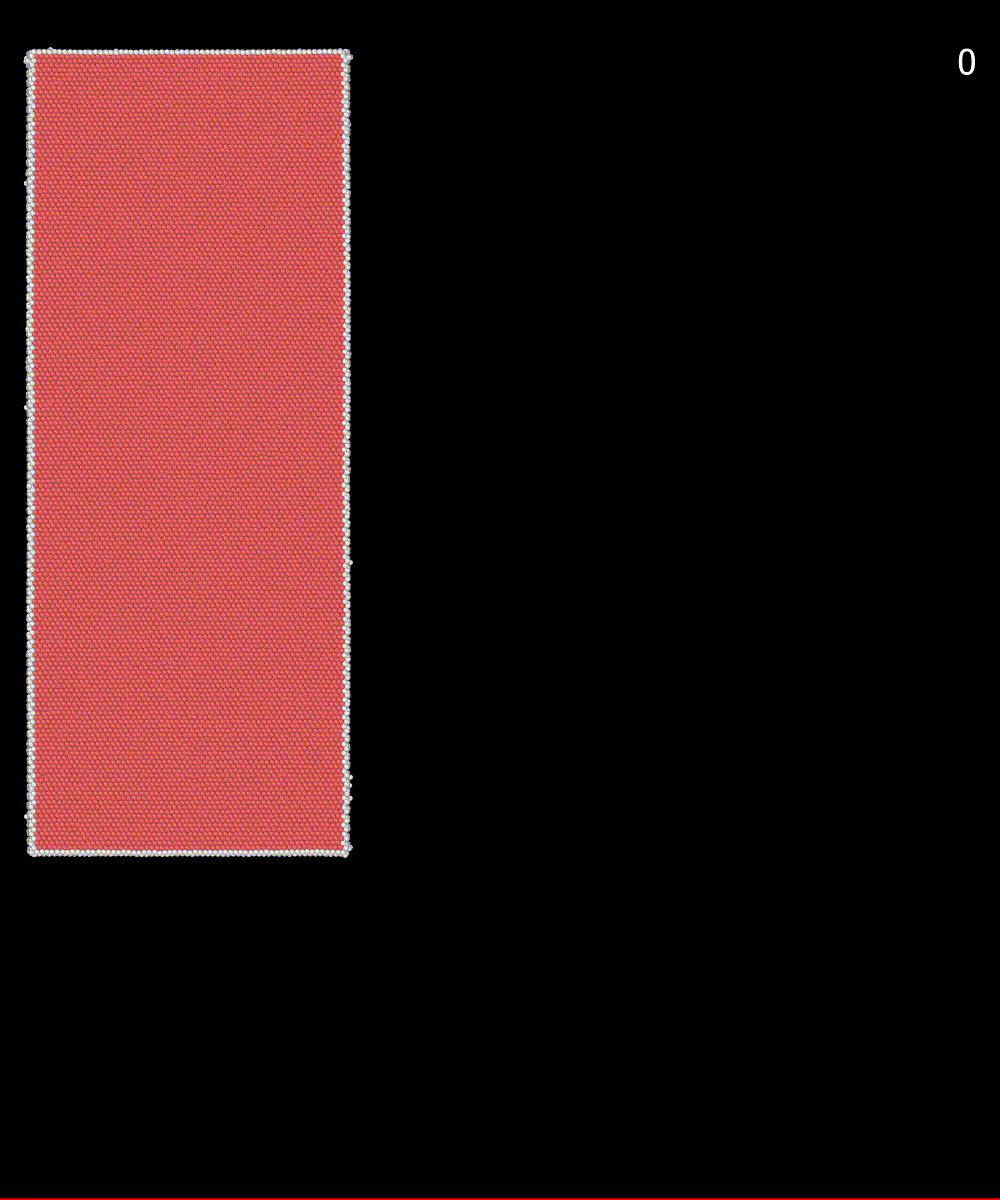

Supplement: mmc4 [file mmc4.gif]

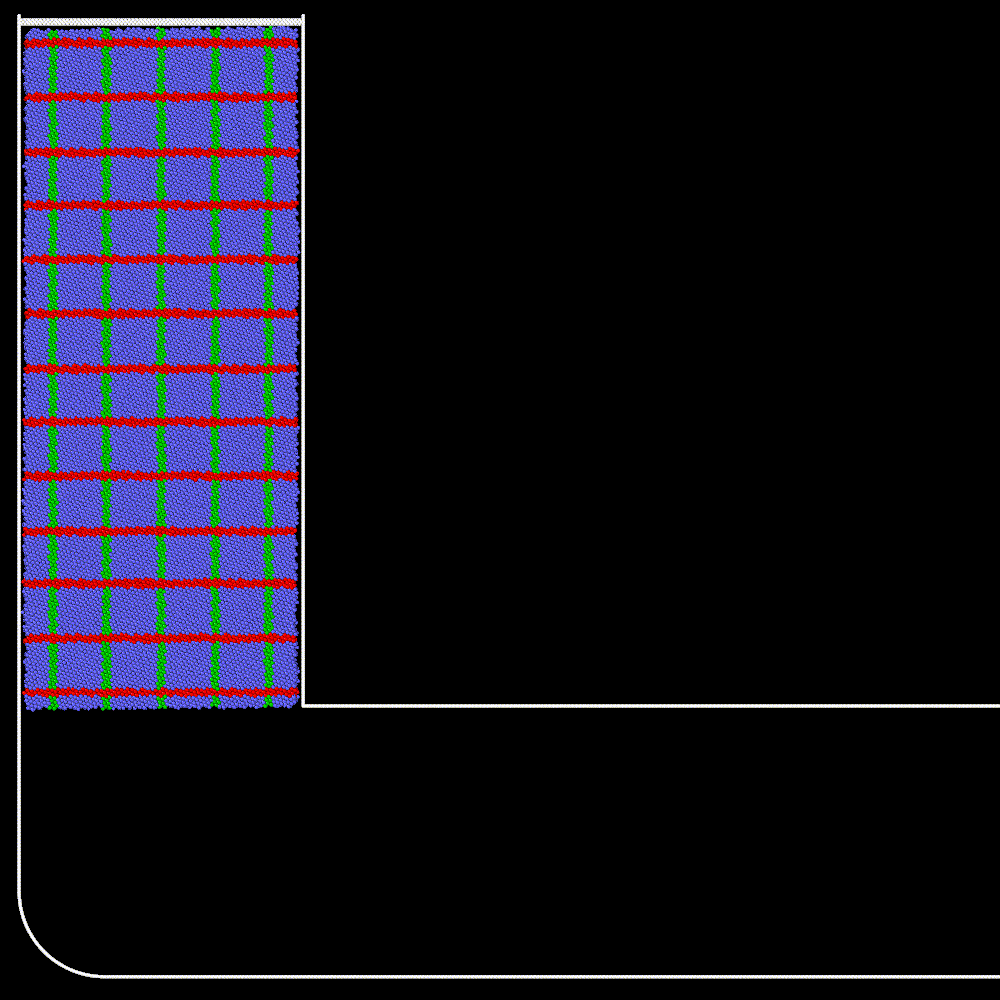

Supplement: mmc5 [file mmc5.gif]

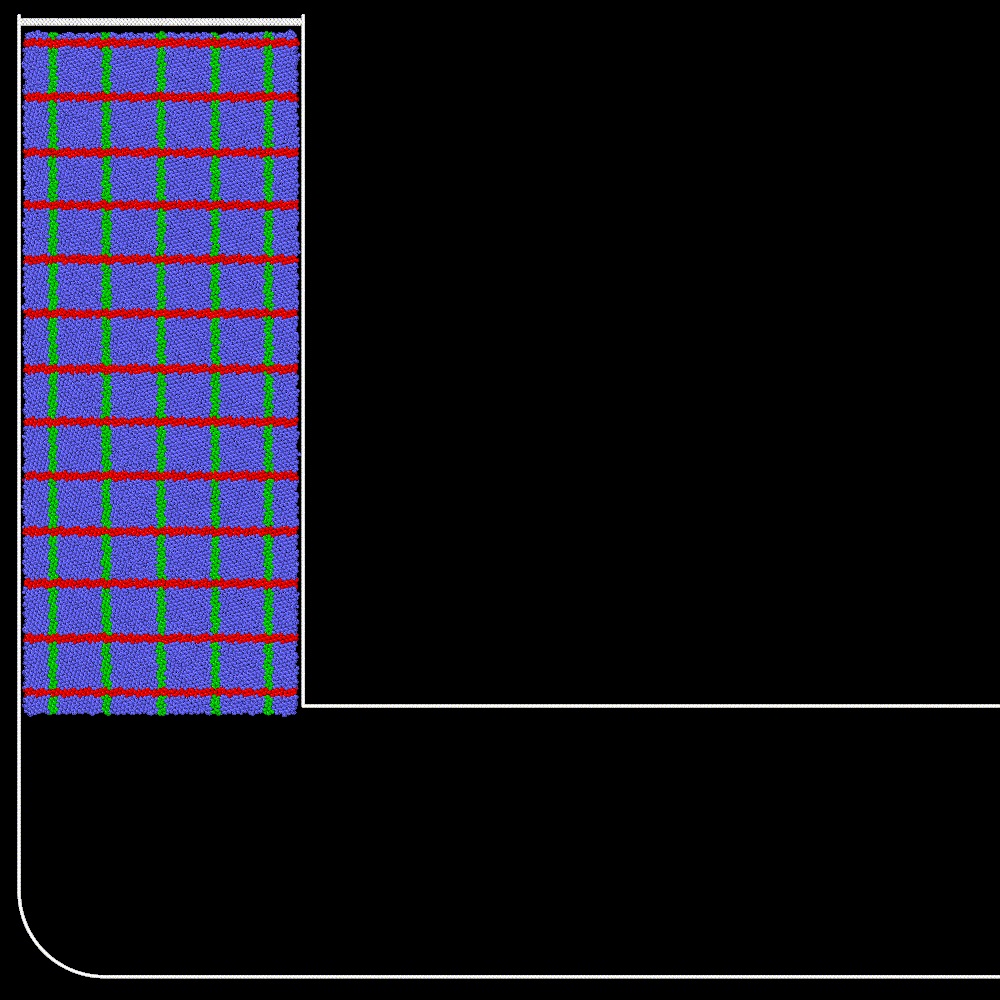

Supplement: mmc6 [file mmc6.gif]

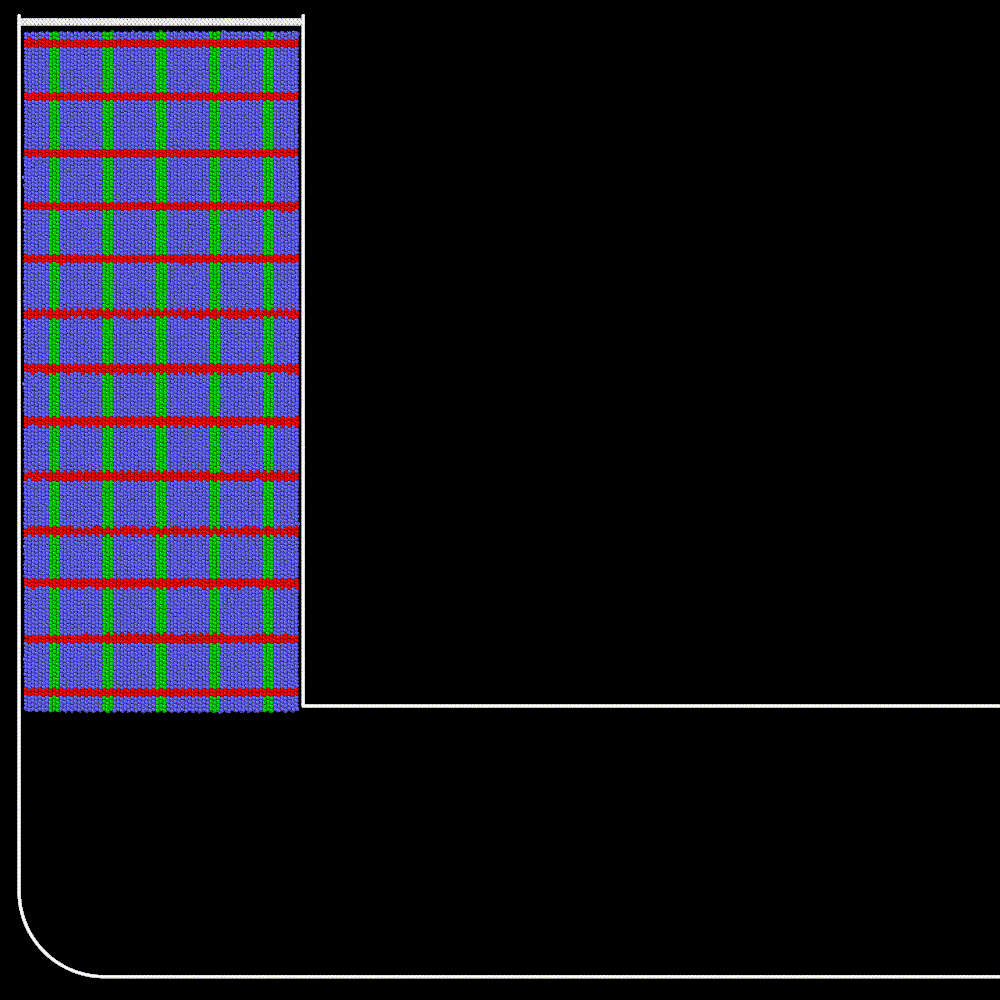

Supplement: mmc7 [file mmc7.gif]

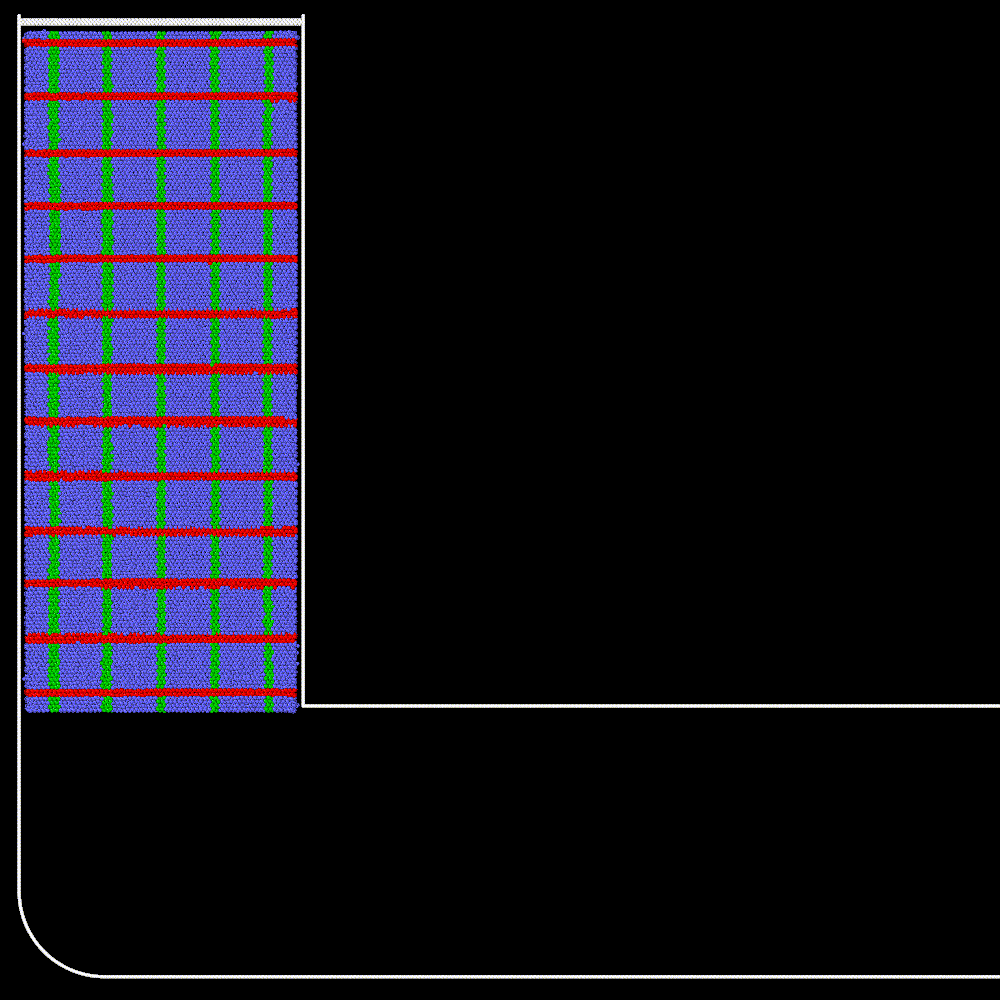

Supplement: mmc8 [file mmc8.gif]
